# Event and Comment

**Published:** 1913-09-15

**Authors:** 


### DENTAL REGISTER.

**Vol. LXVII. September 15, 1913. No. 9**

### EVENT AND COMMENT.

THE NATIONAL MEETING. The Annual Meeting of the National Dental Association held this year at Kansas City was the last meeting to be held under the old constitution. From this time and on we hope the National Association will be in fact as well as in name, a more truly representative body than it has ever pretended to be in its past history. Every Dentist in the United States who cares enough for his profession to identify himself with his local society can be a member of the National Society. Enough States joined at the last meeting to insure a membership of approximately 15000. Whether or not all of these members ever attend a meeting of the National they will have a personal and official representative to speak and act for them and they will receive the complete transactions of the doings of the Association. No one should be so sanguine as to expect that everything will be done in a perfect manner or to our universal satisfaction, but it seems reasonable that a more representative body would at least accomplish more for the profession than one made up of voluntary memberships, as was the case in the older organizations.

One of the best and most promising actions of the new organization was the taking up the matter of scientific research on a plan that promises to bring definite results in the very near future. The Committee having this matter in charge has already started work in several of our research laboratories under the direction of men of experience, which undoubtedly will be fruitful of good results, and will probably be further augmented as the fund becomes available.

It is the plan of the Association to begin the publication of a journal of its own as soon as the necessary funds are in hand. The plan is to publish all the proceedings of the Association as well as papers on scientific subjects by special Contributors and at least some of the component societies papers, and a copy of the journal will be supplied to every member of the Association.

Dr. Homer C. Brown, of Columbus, was elected president; and Dr. Otto U. King of Huntington, Ind, was elected Secretary. The next meeting will be held at Rochester, N. Y., July 14th to the 17th.

THE PACIFIC DENTAL GAZETTE'S NEW EDITOR. Dr. Julio Endelman succeeds Dr. Frank L. Platt, who for many years has so ably edited this interesting Journal. Dr. Endelman has had good training for the work and he should prove a worthy successor to Dr. Platt. Dr. Endelman was for many years associated with Dr. Kirk on the Dental Cosmos, and will not lack practical experience. For several years he had charge of the department of foreign literature which he edited with exceptional care and rare discernment. We predict a successful career for this journal as the publishers are aggressive business men and they promise the new editor their cordial support and give him absolute control of the literary pages. It may be that the handicap of being a trade journal will influence some of the professional support that the journal should have against its best success, but we shall expect Dr. Endelman to overcome this by his skill and ability.

DENTAL OFFICE EQUIPMENT. The question of how to equip a dental office is just at this seaon one that is giving many a young graduate much trouble to decide just how he can manage to buy a respectable and efficient outfit with a small amount or no money at all. Fortunately our dealers have faith in the future of a young man who has made a decent record in college and are willing to take a chance on his ability to pay out in a reasonable time. We recently saw an office that would certainly discourage most young men. There was everything that money could buy and the engenuity of the dentist could suggest in the way of furnishings and equipment. The best part of this particular office is that the work done there is on a par with the equipment. We have in mind another office where good work is not done; the equipment is practically all home made and the laboratory is a veritable junk shop of appliances. The room in the first place is entirely too small for a decent laboratory, and it is packed full of every kind of an appliance that its owner could find time to make or that he could buy. It reminded me of the veritable shoemakers work bench and was kept in the same proverbial disorder. No one can do a decent piece of work with such an outfit, as most of his time will be spent in finding the tools to work with. We would suggest the purchasing of the essential equipment of the best possible quality in the way of efficiency rather than for display and then the additional articles desired can be added when their necessity has been fully demonstrated. Above all avoid untried new appliances.

